# Unique S-scheme heterojunctions in self-assembled TiO_2_/CsPbBr_3_ hybrids for CO_2_ photoreduction

**DOI:** 10.1038/s41467-020-18350-7

**Published:** 2020-09-14

**Authors:** Feiyan Xu, Kai Meng, Bei Cheng, Shengyao Wang, Jingsan Xu, Jiaguo Yu

**Affiliations:** 1 State Key Laboratory of Advanced Technology for Materials Synthesis and Processing, Wuhan University of Technology, Wuhan, 430070 P.R. China; 2 Foshan Xianhu Laboratory of the Advanced Energy Science and Technology Guangdong Laboratory, Xianhu Hydrogen Valley, Foshan, 528200 P.R. China; 3 College of Science, Huazhong Agricultural University, Wuhan, 430070 P.R. China; 4 School of Chemistry and Physics & Centre for Materials Science, Queensland University of Technology, Brisbane, QLD 4001 Australia

**Keywords:** Photocatalysis, Photocatalysis, Quantum dots

## Abstract

Exploring photocatalysts to promote CO_2_ photoreduction into solar fuels is of great significance. We develop TiO_2_/perovskite (CsPbBr_3_) S-scheme heterojunctions synthesized by a facile electrostatic-driven self-assembling approach. Density functional theory calculation combined with experimental studies proves the electron transfer from CsPbBr_3_ quantum dots (QDs) to TiO_2_, resulting in the construction of internal electric field (IEF) directing from CsPbBr_3_ to TiO_2_ upon hybridization. The IEF drives the photoexcited electrons in TiO_2_ to CsPbBr_3_ upon light irradiation as revealed by in-situ X-ray photoelectron spectroscopy analysis, suggesting the formation of an S-scheme heterojunction in the TiO_2_/CsPbBr_3_ nanohybrids which greatly promotes the separation of electron-hole pairs to foster efficient CO_2_ photoreduction. The hybrid nanofibers unveil a higher CO_2_-reduction rate (9.02 μmol g^–1^ h^–1^) comparing with pristine TiO_2_ nanofibers (4.68 μmol g^–1^ h^–1^). Isotope (^13^CO_2_) tracer results confirm that the reduction products originate from CO_2_ source.

## Abstract (web summary)

Rational design and fabrication of high-performance photocatalyst is of great importance for CO_2_ reduction into solar fuel. Here, the authors demonstrate that S-scheme heterojunction TiO_2_/CsPbBr_3_ photocatalyst exhibits enhanced CO_2_ photoreduction activity.

## Introduction

The depletion of fossil fuels and continuous CO_2_ emissions have caused emerging global energy and environmental crises^1–5^. The photoreduction of CO_2_ into renewable fuels with solar energy is recognized as a potential solution to solve above issues^6–10^. As a chemically inert, nontoxic and earth-abundant photocatalyst, TiO_2_ is supposed to be proverbially utilized for CO_2_ photoreduction^11–13^. However, like the majority of unitary photocatalysts, the photocatalytic efficiency of TiO_2_ is still far away from the practical requirements largely due to its rapid electron–hole recombination^14,15^. Hybridizing TiO_2_ with another semiconductor with a suitable band structure is a widely adopted strategy to tackle this issue owing to the efficient separation of photoinduced electron–hole pairs^16–20^. Therefore, it is of significance to explore or design a TiO_2_-based heterojunction to improve the photocatalytic CO_2_ reduction performance.

CsPbBr_3_, a typical material of halide perovskites, has attracted significant scientific interest in optoelectronic applications owing to its outstanding properties, including narrow photoemission, high photoluminescence quantum yield, tunable bandgap, and competing optoelectronic properties^21–24^. Inspired from the achievements in optoelectronic applications, CsPbBr_3_ is a potential candidate for conducting efficient photocatalysis^25,26^. CsPbBr_3_ quantum dots (QDs) have recently been hybridized with 2D graphene oxide^27^ and porous g-C_3_N_4_^28^ for CO_2_ photoreduction. Nevertheless, in these cases, the electrons in the conduction band of CsPbBr_3_ transferred into graphene and g-C_3_N_4_, forming Schottky and type-II heterojunctions, respectively, sacrificing the reduction ability of the photoinduced electrons despite achieving better charge separation. Very recently, an S-scheme heterojunction composed of two n-type semiconductors has been proposed^29,30^. The transfer path of photogenerated charge carriers at interfaces is like an “S” figure, enabling the heterojunctions to have the highest redox ability. The S-type charge transportation correlates with the band bending and internal electric field (IEF) at the junction. The n-type nature and remarkably different work functions of TiO_2_ and CsPbBr_3_ suggest a high possibility of forming S-scheme TiO_2_/CsPbBr_3_ heterojunctions. Up to now, however, constructing perovskite CsPbBr_3_ with TiO_2_, an emerging photoactive material and the most widespread photocatalyst, for efficient CO_2_ photoreduction has not yet been reported.

Herein, we report on a unique TiO_2_/CsPbBr_3_ S-scheme heterojunction built by electrostatic self-assembly of TiO_2_ nanofibers and CsPbBr_3_ QDs for boosted photocatalytic CO_2_ reduction. TiO_2_ nanofibers show no aggregation upon dispersion in solution and thereby retain their phototactically active sites exposed on the surface. Meanwhile, randomly stacked TiO_2_ nanofibres readily form a loose network, facilitating the adsorption–desorption and transportation of reactants and products. More importantly, the TiO_2_ nanofibres are composed of small nanocrystals, possessing interparticle voids and rough surface, which make TiO_2_ nanofibres an ideal host to anchor CsPbBr_3_ QDs. Experimental study and density functional theory (DFT) calculation verify the presence of IEF in the unique TiO_2_/CsPbBr_3_ heterojunction, which separate photoinduced charge carriers more efficiently. We argue the formation of the S-scheme charge transfer route at TiO_2_/CsPbBr_3_ interfaces upon light irradiation. The obtained TiO_2_/CsPbBr_3_ heterojunction shows a superior activity for reducing CO_2_ into solar fuels under UV–visible-light irradiation. This work provides a point of view in TiO_2_-based photocatalyst for efficient CO_2_ photoreduction driven by the S-scheme electron transfer route.

## Results and discussion

### Characterization of as-prepared CsPbBr_3_ QDs

**Fig. 1:**
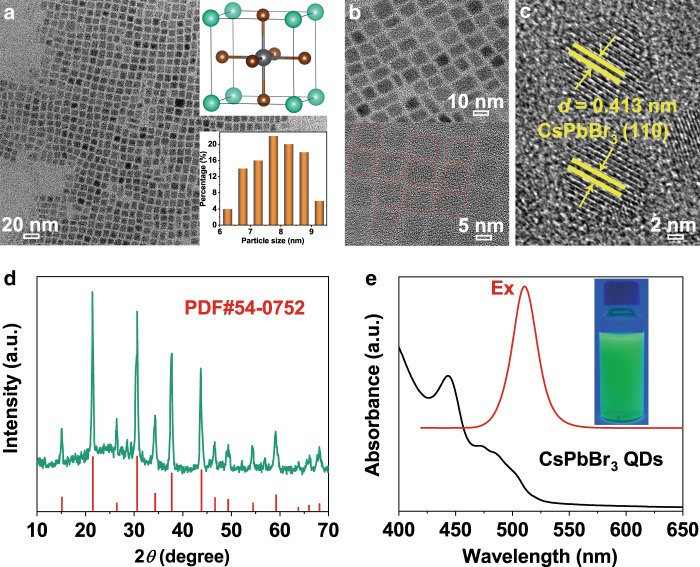
Characterization of CsPbBr_3_ QDs. **a**, **b** Transmission electron microscopy (TEM) image and corresponding size distribution (lower right inset of panel **a**), the geometrical structure (upper right inset of panel **a**), **c** high-resolution TEM (HRTEM) image, **d** X-ray diffraction (XRD) pattern, and **e** UV–vis absorption (black line) and PL emission (red line). Inset shows the photograph of CsPbBr_3_ QDs colloidal solutions in hexane under UV light of 365 nm.

Transmission electron microscopy (TEM) images with different magnifications are shown in Fig. 1a, b. The CsPbBr_3_ QDs were of nanocubes with a size of 6–9 nm (inset in Fig. 1a). High-resolution TEM (HRTEM) image (Fig. 1c) showed lattice spacings of 0.413 nm, corresponding to the (110) facets of CsPbBr_3_. As-prepared CsPbBr_3_ QDs were of cubic phase (JCPDS No. 54-0752) as revealed by X-ray diffraction (XRD) pattern (Fig. 1d). The UV–vis absorption spectrum of CsPbBr_3_ QDs revealed strong bands at 450 and 500 nm (Fig. 1e). The corresponding photoluminescence (PL) spectrum unfolded a narrow emission at 520 nm, agreeing with previous reports^21,31^. Accordingly, the QDs solution showed a bright green fluorescence under 365 nm UV light (inset of Fig. 1e).

### Characterization of TiO_2_/CsPbBr_3_ heterojunction

**Fig. 2:**
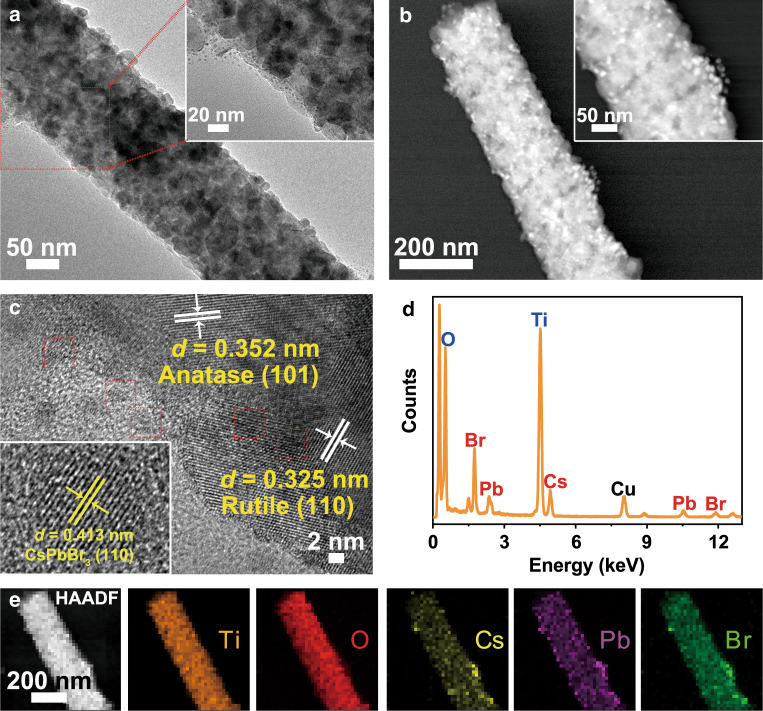
Morphology and structure of TiO_2_/CsPbBr_3_ heterojunction. **a**–**c** Transmission electron microscopy (TEM), STEM, and high-resolution TEM (HRTEM) images of TC2, **d** EDX spectrum of TC2, and **e** high-angle annular dark-field (HAADF) image and EDX elemental mappings of Ti, O, Cs, Pb, and Br elements in TC2.

The TiO_2_/CsPbBr_3_ heterojunction was synthesized via electrostatic self-assembly of TiO_2_ nanofibers and CsPbBr_3_ QDs. Moreover, the minimization of the surface energy of the QDs should also be responsible for their adsorption to the TiO_2_ nanofibers. The TiO_2_/CsPbBr_3_ hybrids were denoted as TC*x*, where T and C denote TiO_2_ and CsPbBr_3_ QDs, respectively; *x* represents the weight percentage of CsPbBr_3_ with respect to TiO_2_. The phase structures of TiO_2_, TC2, and TC4 were determined via XRD analysis (Supplementary Fig. 1). TiO_2_ nanofibers showed intensive reflections belonging to anatase (JCPDS No. 21-1272) and rutile (JCPDS No. 21-1276) phases. TC2 showed a similar XRD pattern with pristine TiO_2_, where the reflections of CsPbBr_3_ QDs cannot be distinguished due to their low content. Apart from the characteristic reflections of TiO_2_, TC4 showed additional reflections at 21.5° and 30.6°, which corresponded to the (110) and (200) planes of CsPbBr_3_ QDs, confirming the formation of TiO_2_/CsPbBr_3_ nanohybrids. The morphology and crystalline phase of pristine TiO_2_ (Supplementary Fig. 2a) exhibited a porous nanofibrous shape with an average diameter of 200 nm. The porous feature was further revealed by the N_2_ sorption isotherms of TC*x* (Supplementary Fig. 3). All the TC*x* samples showed similar pore size distributions with a wide range of 10–20 nm, much larger than the size of CsPbBr_3_ QDs (6–9 nm). The resultant specific surface areas (*S*_BET_), pore volumes (*V*_p_), and average pore sizes (*d*_p_) presented a volcano shape with increasing the loading of CsPbBr_3_ QDs (Supplementary Table 1). At a low QDs loading (<2 wt.%), TC*x* showed an increased *S*_BET_ and reached the maximum value at TC2 because the low filling enables QDs to deposit onto the inner wall of TiO_2_ mesopores. Such island-like QDs on the inner wall contribute additional specific surface area for the hybrid. When the QDs loading was further increased, QDs would aggregate in TiO_2_ mesopores and the island-like distribution vanished, which thereby resulted in a decrease of *S*_BET_. The HRTEM image (Supplementary Fig. 2b) showed clear lattice spacings of 0.352 and 0.325 nm, corresponding to anatase (101) and rutile (110) d-spacings, respectively. After the assembling process, the QDs were uniformly deposited on the TiO_2_ nanofibers (Fig. 2a, b). The lattice spacings of anatase and rutile phase TiO_2_, as well as CsPbBr_3_ QDs, appeared in the HRTEM image, as shown in Fig. 2c, confirming the formation of TiO_2_/CsPbBr_3_ nanohybrids. The energy-dispersive X-ray spectroscopy (EDX) spectrum of TC2 (Fig. 2d) revealed the existence of Cs, Pb, and Br apart from the dominant Ti and O elements. All the elemental mappings overlapped perfectly (Fig. 2e). Fourier-transform infrared (FTIR) spectra showed the presence of (Ti)–OH on TiO_2_ and organic residues on QDs (Supplementary Fig. 4a, b)^32^. The (Ti)–OH signal weakened upon QDs deposition owing to the shielding effect of QDs. All the results confirmed the successful electrostatic assembly of TiO_2_ nanofibers and CsPbBr_3_ QDs.

The optical absorption of the samples was investigated by UV–vis diffuse reflectance spectrometer (DRS) (Supplementary Fig. 5a). The absorption edges of pristine TiO_2_ nanofibers and CsPbBr_3_ QDs were located at 400 and 550 nm, corresponding to the bandgap energy of 3.10 and 2.24 eV, respectively (Supplementary Fig. 5b). In comparison with pristine TiO_2_, TC*x* showed two obvious absorption edges belonging to TiO_2_ and CsPbBr_3_ QDs, and exhibited slightly enhanced UV and visible-light harvesting when increasing the amount of CsPbBr_3_ QDs owing to the strong light-harvesting capability of perovskite QDs. Note that the calculated bandgap energy of TiO_2_ and CsPbBr_3_ in TC4 was different from their intrinsic bandgap, implying that there exist electrostatic attraction and interaction between TiO_2_ and CsPbBr_3_ during the hybridization.

**Fig. 3:**
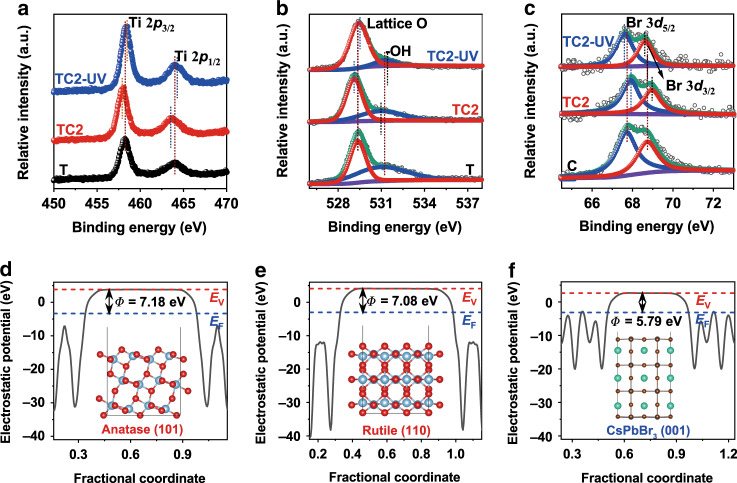
Electron transfer between TiO_2_ and CsPbBr_3_ quantum dots (QDs). In-situ and ex-situ X-ray photoelectron spectroscopy (XPS) spectra of **a** Ti 2*p*, **b** O 1*s*, and **c** Br 3*d* of TiO_2_, CsPbBr_3_, and TC2. In-situ XPS spectra were collected under UV–vis light irradiation. The electrostatic potentials of **d** anatase TiO_2_ (101), **e** rutile TiO_2_ (110), and **f** CsPbBr_3_ (001) facets. The blue, red, green, gray, and brown spheres stand for Ti, O, Cs, Pb, and Br atoms, respectively. Blue and red dashed lines indicate the Fermi and vacuum energy levels.

X-ray photoelectron spectroscopy (XPS) was further performed to explore the chemical states of the resultant samples. The survey XPS spectrum (Supplementary Fig. 6a) showed the presence of Cs, Pb, and Br elements within TC2, as well as Ti and O. The ex-situ Ti 2*p* XPS spectra of TiO_2_ and TC2 (Fig. 3a) showed symmetrical Ti 2*p* doublets of Ti^4+^ ions. The O 1*s* XPS spectra (Fig. 3b) revealed the presence of lattice oxygen (529.3 eV) and –OH surface group (531.2 eV). Interestingly, TC2 showed a weaker XPS signal of –OH than pristine TiO_2_, which was also attributed to an increase of QDs over TiO_2_ nanofiber surface and was in agreement with the above FTIR results. The Br 3*d*-binding energies (BEs) of CsPbBr_3_ QDs were 67.8 and 69.8 eV, corresponding to Br 3*d*_5/2_ and Br 3*d*_3/2_, respectively (Fig. 3c). Noticeably, the BEs of Ti 2*p* and O 1*s* in TC2 were shifted by 0.2 eV toward a lower BE in comparison with those of pristine TiO_2_, while the Cs 3*d*, Pb 4*f* (Supplementary Fig. 6c, d) and Br 3*d* BEs of TC2 became more positive as compared with those of QDs, indicating that the electrons transferred from CsPbBr_3_ QDs to TiO_2_ upon hybridization due to the difference of their work functions. Such electron transfer created an IEF at interfaces pointing from QDs to TiO_2_, facilitating the construction of S-scheme TiO_2_/CsPbBr_3_ heterojunction without any redox mediator, which would efficiently separate the charge carriers and thus promote the CO_2_ photoreduction^33–35^.

Work function (*Φ*), as another important parameter to study the electron transfer within duplicate semiconductor heterostructures, can be estimated from the energy difference of vacuum and Fermi levels according to the electrostatic potential of a material. As shown in Fig. 3d–f, the work function of anatase TiO_2_ (101), rutile TiO_2_ (110), and CsPbBr_3_ QDs (001) were 7.18, 7.08, and 5.79 eV, respectively, indicating that both anatase and rutile TiO_2_ have lower Fermi levels than CsPbBr_3_ QDs. When they contacted with each other, electrons would flow from CsPbBr_3_ to anatase and/or rutile TiO_2_ to enable the phases at the same Fermi level and definitely created an IEF at TiO_2_/CsPbBr_3_ interfaces. These results were absolutely consistent with above ex-situ XPS results and beneficial to the charge separation and CO_2_ photoreduction activity.

### CO_2_ photoreduction activity of TiO_2_/CsPbBr_3_ hybrids

**Fig. 4:**
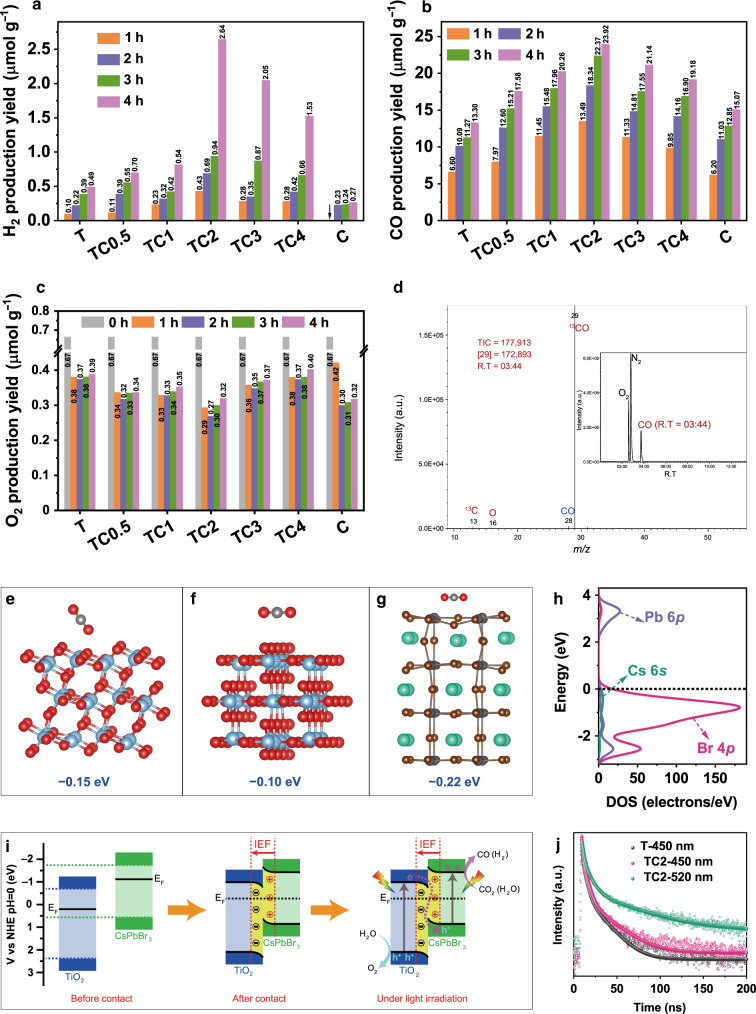
CO_2_ photoreduction performance and the photocatalytic mechanism of S-scheme heterojunction. Photocatalytic activities of CO_2_ reduction over TiO_2_, TC*x*, and CsPbBr_3_ quantum dots (QDs) during 4-h experiment performed under UV–vis light irradiation: time course of **a** H_2_, **b** CO, and **c** O_2_ production yields. The initial O_2_ concentrations were normalized. **d** Mass spectra of ^13^CO and total ion chromatography (inset) over TC2 in the photocatalytic reduction of ^13^CO_2_. Optimized structures of CO_2_ molecule adsorbed on **e** anatase TiO_2_ (101), **f** rutile TiO_2_ (110), and **g** CsPbBr_3_ (001) facets. The blue, red, green, gray, and brown spheres stand for Ti, O, Cs, Pb, and Br atoms, respectively. **h** The DOS of CsPbBr_3_. **i** Schematic illustration of TiO_2_/CsPbBr_3_ heterojunction: internal electric field (IEF)-induced charge transfer, separation, and the formation of S-scheme heterojunction under UV–visible-light irradiation for CO_2_ photoreduction. **j** Time-resolved photoluminescence (TRPL) spectra of TiO_2_ (T) and TC2 at emission wavelengths of 450 and 520 nm, respectively.

The CO_2_ photoreduction activity of resultant samples was measured in a closed gas-circulation system (Supplementary Fig. 7) with a Quartz and Pyrex glass hybrid reaction cell (Supplementary Fig. 8) and the photocatalytic reduction products consisted of a majority of CO and a small amount of H_2_. The original chromatograms for the reduction of CO_2_ on sample TC2 are shown in Supplementary Fig. 9. Control experiments (Supplementary Fig. 10 and Table 2) showed that neither H_2_ nor CO was detected in the dark or in the absence of CO_2_, suggesting that the light irradiation and input CO_2_ were indispensable for the photocatalytic reaction. As shown in Fig. 4a, b, pristine TiO_2_ and CsPbBr_3_ QDs exhibited relatively lower production rates of H_2_ (0.12 and 0.06 μmol g^–1^ h^–1^, respectively) and CO (4.68 and 4.94 μmol g^–1^ h^–1^, respectively), resulting from the rapid charge recombination. Note that the H_2_ and CO productions were greatly enhanced with increased loading of QDs, and the generation of CO reached a maximum rate (9.02 μmol g^–1^ h^–1^) with a relatively high selectivity (95%) over TC2, due to the efficient charge separation of TiO_2_/CsPbBr_3_ heterostructure. Further increasing CsPbBr_3_ QDs amount would be detrimental to the photocatalytic activity (e.g., TC3 and TC4), because the overloading of CsPbBr_3_ could shield the light absorption of TiO_2_ and decrease *S*_BET_ of the nanohybrids. Interestingly, with the reaction time went on, the amount of O_2_ decreased first and then increased, as shown in Fig. 4c. The initial O_2_ in the system came from the input high-purity CO_2_. In the first two hours of photocatalytic CO_2_ reduction, the fresh materials exhibit relatively strong reactivity of photoreduction. As a competitive reaction to CO_2_ reduction, the consumption rate of O_2_ (O_2_ + e^–^ → ·O_2_^–^) was much higher than the production rate at the initial 2 h, while in the following 2 h, the production rate of O_2_ was higher than the consumption rate, the total amount of oxygen and the ratio of oxygen:nitrogen have increased to a certain extent.

The recyclability and stability of TC2 for CO_2_ photoreduction were investigated (Supplementary Fig. 11). After four times cycles, the decay of photocatalytic production yields of H_2_ and CO were hardly perceptible. To evaluate the photostability of the nanohybrids, we have characterized the recycled photocatalyst using XRD, TEM, XPS, and FTIR. As shown in the XRD pattern (Supplementary Fig. 12a), the used photocatalyst showed no detectable phase change. The TEM image confirms that the QDs did not show obvious aggregation after cycled photocatalytic reactions, and the morphology was well maintained (Supplementary Fig. 12b). The chemical states of the used photocatalyst were also consistent with those of the fresh one, as examined by XPS (Supplementary Fig. 13). The FTIR spectra of TC2 before and after reaction were presented in Supplementary Fig. 14. The characteristic absorbance bands of the aliphatic species from QDs showed no obvious variation, implying that the capping agent of QDs was stable and was not decomposed during the photocatalytic CO_2_ reduction.

To determine the origin of CO_2_ photoreduction products, we performed an isotope-labeled carbon dioxide (^13^CO_2_) photocatalytic reduction over TC2. Since the amount of products without photosensitizer and hole sacrificial agent was beyond the detection limit of mass spectrometry detector, we added tris(2,2’-bipyridyl)ruthenium(II) chloride hexahydrate ([Ru^II^(bpy)_3_]Cl_2_·6H_2_O)^36^ and 1,3-dimethyl-2-phenyl-2,3-dihydro-1H-benzo[d]imidazole (BIH)^37^ into the system to promote the photocatalytic activity, which behaved as the photosensitizer and hole sacrificial agent, respectively. In this case, the production yields of H_2_ and CO were significantly enhanced (Supplementary Fig. 15 and Table 2) and readily detected by gas chromatography–mass spectrometer (GC-MS). As shown in Fig. 4d, the total ion chromatographic peak ~3.44 min corresponded to CO, which produced three signals in the mass spectra. The main MS signal at *m/z* = 29 belonged to ^13^CO and the others (^13^C at *m/z* = 13 and O at *m/z* = 16) corresponded to the fragments of ^13^CO, confirming that the CO product exactly originated from the CO_2_ photoreduction over TiO_2_/CsPbBr_3_^38,39^. In addition, the total ion chromatographic peaks ~2.36 and 2.48 min can be assigned to the O_2_ and N_2_, respectively (Supplementary Fig. 16).

The CO_2_ adsorption of a photocatalyst is an essential step for CO_2_ photoreduction^40^. Figure 4e–g compared the optimized models of one CO_2_ molecule adsorbed on anatase TiO_2_ (101), rutile TiO_2_ (110), and CsPbBr_3_ (001) surfaces. Clearly, the adsorption energy (*E*_ads_) of CO_2_ onto CsPbBr_3_ (−0.22 eV) was more negative than that onto anatase and rutile TiO_2_ (−0.15 and −0.10 eV), which suggests that CO_2_ molecules adsorbed on CsPbBr_3_ is more stable than on TiO_2_. The results also indicate that CsPbBr_3_ QDs were in favor of the adsorption of CO_2_ molecules and the photocatalytic CO_2_ reduction.

To further explore the photocatalytic mechanism, the band structures of TiO_2_ and CsPbBr_3_ QDs were investigated. The valence band (VB) potential was obtained by analyzing the VB XPS spectra. As shown in Supplementary Fig. 17a, b, the energy level of valence band maximum (VBM) of TiO_2_ and CsPbBr_3_ is 2.39 and 1.03 eV, respectively. Mott–Schottky (M–S) curves showed that TiO_2_ and CsPbBr_3_ were of n-type semiconductors and had flat-band potentials of 0.01 eV and −0.51 eV (vs. NHE), respectively (Supplementary Fig. 17c, d). Thus, the band structures of TiO_2_ and CsPbBr_3_ QDs can be derived, and the positions of VBM and conduction band minimum (CBM) of TiO_2_ and CsPbBr_3_ are shown in Supplementary Fig. 17f.

### Photocatalytic mechanism of S-scheme heterojunction

From the above analysis, the superior photoreduction activity was ascribed to the stronger CO_2_ adsorption of CsPbBr_3_ QDs and the formation of S-scheme heterojunction between TiO_2_ and CsPbBr_3_ QDs. As revealed by the above ex-situ XPS and DFT analyses, TiO_2_ has a lower Fermi level than CsPbBr_3_ QDs before they contact. Upon hybridization, the electrons preferred to flow from CsPbBr_3_ QDs to TiO_2_, which created an IEF at TiO_2_/CsPbBr_3_ interfaces pointing from CsPbBr_3_ to TiO_2_ and bent the energy bands of TiO_2_ and CsPbBr_3_. Upon photoexcitation, the VB electrons of TiO_2_ and CsPbBr_3_ jumped to their CBs. Driven by the interfacial IEF and bent bands, the photogenerated electrons in TiO_2_ CB spontaneously slid toward CsPbBr_3_ and recombined with the holes in CsPbBr_3_ VB. The electron-rich CsPbBr_3_ QDs then acted as active sites and donated electrons to activated CO_2_ molecules for producing H_2_ and CO. Noted that Pb was the active site for CO_2_ photoreduction since the CB of CsPbBr_3_ was mainly consisted of Pb 6*p* orbitals as evidenced by the density of states (DOS) of CsPbBr_3_ (Fig. 4h). Clearly, the transportation of photoinduced charge carriers follows a slide-like pathway, which implies the presence of S-scheme heterojunction between TiO_2_ and CsPbBr_3_ QDs. This unique S-scheme charge transfer efficiently separated the photoinduced electron–hole pairs and meanwhile remained the high redox ability of electrons in CsPbBr_3_ CB and holes in TiO_2_ VB, respectively. The S-scheme heterostructure of TiO_2_/CsPbBr_3_ QDs along with the charge transfer and separation is illustrated in Fig. 4i. Such an S-scheme charge transfer route was strongly evidenced by the in-situ XPS spectra measured under light irradiation. As revealed in Fig. 3a–c and Supplementary Fig. 6c, d, the BEs of Ti 2*p* and O 1*s* for TC2 under light irradiation shifted positively by 0.3 eV with reference to those in the corresponding ex-situ spectra. Conversely, the BEs of Cs 3*d*, Pb 4*f*, and Br 3*d* of TC2 shifted negatively by 0.5 eV. The BE shifts unequivocally proved that the photoexcited electrons in TiO_2_ CB transferred to CsPbBr_3_ QDs VB under light irradiation, following an S-scheme pathway, which supported the proposed photocatalytic mechanism.

It is worth mentioning that the TiO_2_ we used consisted of both anatase and rutile phases, and the charge transfer between the two phases may take place as a result of forming homojunction. As evidenced by DFT results (Fig. 3d, e), the work function of anatase TiO_2_ (101) was larger than that of rutile TiO_2_ (110), indicating that electrons would flow from rutile to anatase and created an IEF at anatase/rutile TiO_2_ interfaces. Driven by the interfacial IEF, the photogenerated electrons in anatase TiO_2_ CB spontaneously slid toward rutile TiO_2_ VB and recombined with the holes in the rutile TiO_2_ VB. Such transportation of photoinduced charge carriers follows an S-like pathway (S-scheme homojunction) between anatase and rutile TiO_2_ (Supplementary Fig. 18), which is consistent with our previous work^41^. When CsPbBr_3_ QDs deposited on TiO_2_ nanofibers, all possible schematic illustrations between anatase TiO_2_, rutile TiO_2_, and CsPbBr_3_ QDs are shown in Supplementary Fig. 19.

To further prove the efficient charge separation of TiO_2_/CsPbBr_3_ S-scheme heterojunction, photoluminescence (PL) emission spectra of the samples were collected (Supplementary Fig. 20). TC2 and TC4 showed a marginally lower PL intensity than TiO_2_, implying that the presence of CsPbBr_3_ QDs efficiently retarded the electron–hole recombination in TiO_2_. To gain a deeper insight into the charge transfer dynamics, the time-resolved photoluminescence (TRPL) spectra of TiO_2_ and TC2 were recorded at emission wavelengths (*E*_W_) of 450 nm and 520 nm (Fig. 4j), corresponding to the maximum fluorescence emissions of TiO_2_ and QDs, respectively. The fitted decay curves disclose the lifetime (*τ*) and percentage (*Rel*.%) of charge carriers (Supplementary Table 3). The short lifetime (*τ*_1_) corresponds to radiative recombination of the carriers (denoted as *τ*_1_-carriers), while the long lifetimes (*τ*_2_ and *τ*_3_) correspond to non-radiative recombination and energy-transfer process^42^. Note that the un-recombined *τ*_1_-carriers will participate in surface photocatalytic reaction. Thus, the decrease of *τ*_1_-carrier percentage implies radiative recombination inhibited. At *E*_W_ = 450 nm, only TiO_2_ showed a fluorescence emission signal. As shown in Supplementary Table 3, TC2 had a lower percentage (36.27%, 450 nm) of *τ*_1_-carriers than pristine TiO_2_ (37.98%, 450 nm), suggesting the radiative recombination over TiO_2_ was inhibited upon QDs deposition due to the formation of S-scheme heterojunction^43,44^. Further, a similar decrease in *τ*_1_-carrier percentage was also observed at *E*_W_ = 520 nm. Notably, TC2 showed longer lifetime than pristine TiO_2_ due to the transfer of the electrons in TiO_2_ CB to QDs VB. Therefore, it is not surprising that the TC2 composite sample exhibited enhanced photocatalytic CO_2_ reduction performance.

The electrochemical impedance spectra (EIS) (Supplementary Fig. 21a) showed the samples with CsPbBr_3_ QDs exhibited smaller semicircle compared to pure TiO_2_ and revealed lower charge-transfer resistance. The polarization curves of TiO_2_ and TC2 under light irradiation (Supplementary Fig. 21b) showed that the overpotential for TC2 was much lower than that of TiO_2_, indicating that TiO_2_/CsPbBr_3_ hybrids presented better reduction capability than that of TiO_2_. These results proved that CsPbBr_3_ QDs, as an emerging semiconductor, could form S-scheme heterojunction with TiO_2_ to promote the electron transfer and separate the electron–hole pairs for efficient CO_2_ photoreduction.

In summary, an S-scheme TiO_2_/CsPbBr_3_ heterojunction synthesizes through an electrostatic assembly method. The resulting TiO_2_/CsPbBr_3_ heterojunction reveals an enhanced activity toward CO_2_ photoreduction under UV–visible-light irradiation due to the IEF-induced, more efficient charge separation between TiO_2_ and CsPbBr_3_. DFT calculations reveal the work function of TiO_2_ was greater than that of CsPbBr_3_, implying electrons transfer from CsPbBr_3_ to TiO_2_ upon hybridization and thus created an IEF at interfaces. The IEF drives photoinduced electrons in TiO_2_ CB to immigrate to CsPbBr_3_ VB as evidenced by in-situ XPS analysis, confirming an S-path of charge transfer. Isotope (^13^CO_2_) tracer results confirm that the reduction products originate from CO_2_ source, instead of any contaminant carbon species. This work provides a point of view in the design of photocatalysts with distinct heterojunctions for efficient photocatalytic CO_2_ reduction.

## Methods

### Synthesis of electrospun TiO_2_ nanofibers

All the chemicals were of analytic grade and purchased from Shanghai Chemical Company. Typically, tetrabutyl titanate (TBT, 2.0 g) and poly(vinyl pyrrolidone) (PVP, 0.75 g, MW = 1,300,000) were mixed with ethanol (10.0 g) and acetic acid (2.0 g) to form a transparent pale-yellow solution after magnetic stirring for 5 h. Afterward, the solution was transferred into a 10-mL syringe in an electrospinning setup with a voltage of 20 kV and a solution feeding rate of 2.5 mL h^–1^. The needle-to-collector distance was 10 cm. The collected TiO_2_ precursor was annealed at 550 °C for 2 h with a heating rate of 2 °C min^–1^ in air.

### Synthesis of perovskite CsPbBr_3_ QDs

Briefly, 130 mg of Cs_2_CO_3_ (0.4 mmol) were mixed with octadecylene (ODE, 6 mL) and oleic acid (OA, 0.5 mL) under stirring in a three-neck flask (25 mL). The mixture was dried at 120 °C for 1 h under vacuum and heated to 150 °C under N_2_ gas to form Cs(oleate) solution, which was stored at room temperature and preheated to 140 °C prior to use. Then 72 mg of PbBr_2_ (0.196 mmol) was mixed with ODE (5.0 mL), oleylamine (0.5 mL), and OA (0.5 mL) in another flask (25 mL), and was dried under vacuum at 105 °C for 0.5 h. The mixture was heated to 170 °C, and Cs(oleate) (0.45 mL) was rapidly injected under vigorously stirring for 5 s. The reaction was quenched by immersing the flask into an ice-water bath. The obtained product was mixed with 3 mL of hexane and centrifuged at 1208 × *g* for 2 min to remove aggregates and large particles. The supernatant was precipitated with acetone and centrifuged at 3355 × *g* for 5 min. As-collected CsPbBr_3_ QDs were re-dispersed in hexane for further use.

### Preparation of TiO_2_/CsPbBr_3_ heterostructures

Typically, 200 mg of TiO_2_ nanofibers were dispersed into 20 mL of hexane. A certain amount of CsPbBr_3_ QDs solution was added into TiO_2_ suspension under vigorous stirring for 2 h. TiO_2_ and CsPbBr_3_ QDs were assembled by electrostatic self-assembly. The mixture was then vacuum-dried at 50 °C for 2 h to form TiO_2_/CsPbBr_3_ heterostructures. The products are labeled as TC*x*, where T and C denote TiO_2_ and CsPbBr_3_ QDs, respectively; *x* is the mass percentage of CsPbBr_3_ QDs.

### Characterization

XRD was performed on a D/Max-RB X-ray diffractometer (Rigaku, Japan) with Cu Kα radiation. TEM images were observed on a Titan G2 60-300 electron microscope equipped with an EDX spectrometer. UV–visible DRS was collected on a Shimadzu UV-2600 UV–visible spectrophotometer (Japan). XPS was performed on a Thermo ESCALAB 250Xi instrument with Al K_α_ X-ray radiation. In-situ XPS was conducted under the same condition, except that UV–visible-light irradiation was introduced. FTIR spectra were recorded with an attenuated total reflectance (ATR) mode on Nicolet iS 50 (Thermo Fisher, USA). The PL emission spectra were collected on a fluorescence spectrophotometer (F-7000, Hitachi, Japan). TRPL spectra were recorded on a fluorescence lifetime spectrophotometer (FLS 1000, Edinburgh, UK) at an excitation wavelength of 325 nm. Electrochemical measurements were conducted on an electrochemical analyzer (CHI660C, CH Instruments, Shanghai). Pt wire, Ag/AgCl (saturated KCl), and 0.5 M Na_2_SO_4_ solution functioned as the counter electrode, reference electrode, and electrolyte, respectively. For the working electrode, 20 mg of TC*x* was ground in 1.0 mL of ethanol and 10 μL of Nafion solution to make a slurry, which was coated onto F-doped SnO_2_-coated (FTO) glass with an exposed area of 1 cm^2^. The FTO electrode was then vacuum-dried at 60 °C for 1 h.

### Photocatalytic CO_2_ reduction

The photocatalytic CO_2_ reduction was performed in a gas-closed system equipped with a gas-circulated pump. The apparatus of the system is shown in Supplementary Fig. 7. Typically, 10 mg of photocatalysts, 30 mL of acetonitrile, and 100 μL of water were added in a Quartz and Pyrex glass hybrid reaction cell (Supplementary Fig. 8). The airtight system was completely evacuated by using a vacuum pump. Then ~80 kPa of high-purity CO_2_ (99.999%) gas was injected. After adsorption equilibrium, the photocatalytic cell was irradiated with a 300 W Xe arc lamp (PLS-SXE300D, Beijing Perfectlight, China), and the reaction system was kept at 10 °C as controlled by cooling water. The CO_2_-reduction products were analyzed on a gas chromatograph (GC-2030, Shimadzu Corp., Japan) equipped with a barrier discharge ionization detector (BID) and a capillary column (Carboxen 1010 PLOT Capillary, 60 m × 0.53 mm). The column was maintained at 35 °C for 15 min. It was then heated to 180 °C at 20 °C min^–1^, and maintained for another 5 min. Helium was the carrier gas with pressure set to 70 kPa. The temperatures of the injector and BID were set to be 150 and 280 °C, respectively. For comparison, 2 mM of tris(2,2′-bipyridyl)ruthenium(II) chloride hexahydrate ([Ru^II^(bpy)_3_]Cl_2_·6H_2_O) and 10 mM of 1,3-dimethyl-2-phenyl-2,3-dihydro-1H-benzo[d]imidazole (BIH) were added into the photocatalytic system (other parameters were unchanged), which behaved as the photosensitizer and hole sacrificial agent, respectively. A series of control experiments were also conducted, and the results are summarized in Supplementary Table 2.

### Isotope-labeling measurement

The isotope-labeling experiment was conducted by using ^13^CO_2_ (isotope purity, 99% and chemical purity, 99.9%, Tokyo Gas Chemicals Co., Ltd.) as the carbon source. Typically, 10 mg of photocatalysts, 2 mM of [Ru^II^(bpy)_3_]Cl_2_·6H_2_O, 10 mM of BIH, 30 mL of acetonitrile and 100 μL of water were loaded into the reaction cell. The protocol of ^13^CO_2_ photoreduction was the same as that mentioned above. The gas products were analyzed by gas chromatography–mass spectrometry (JMS-K9, JEOL-GCQMS, Japan and 6890 N Network GC system, Agilent Technologies, USA) equipped with the column for detecting the products of ^13^CO (HP-MOLESIEVE, 30 m × 0.32 mm × 25 μm). Helium was used as carrier gas. The column was maintained at 60 °C for 20 min, and the flow of the carrier was 0.5 ml L^–1^. The temperatures of the injector, EI source, and the GCITF were set to be 200, 200, and 250 °C, respectively.

## Supplementary information

Supplementary Information

Peer Review File

## Data Availability

All data are available from the corresponding author on request. Source data are provided with this paper. Source data are also available in figshare with the identifier 10.6084/m9.figshare.12715484.

## Source data

Source Data
