# Supplementary material for: Unique S-scheme heterojunctions in self-assembled TiO2/CsPbBr3 hybrids for CO2 photoreduction
Source: Nat Commun. 2020 Sep 14;11:4613. doi: 10.1038/s41467-020-18350-7 (PMC7490390; doi:10.1038/s41467-020-18350-7)
Supplement: Supplementary file 1 — Supplementary Information [file 41467_2020_18350_MOESM1_ESM.pdf]

Supplementary Information

Unique S-scheme heterojunctions in self-assembled TiO_2/$CsPbBr_3$ hybrids for CO_2 photoreduction

Xu *et al.*

Supplementary Figures

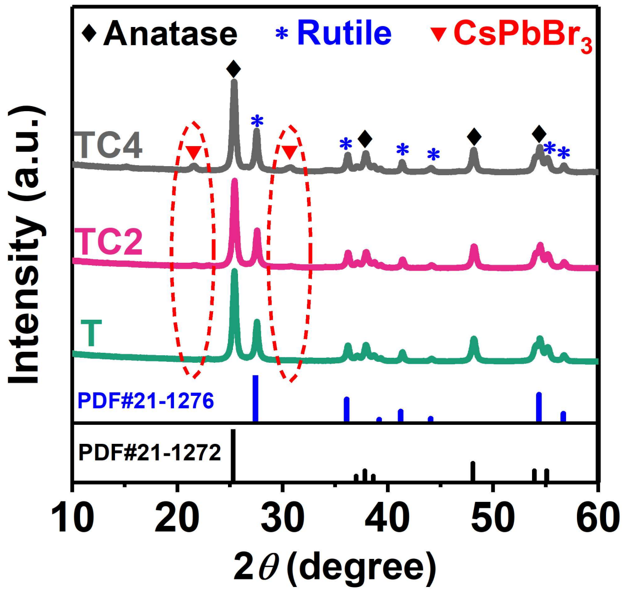

Supplementary Figure 1. X-ray diffraction (XRD) patterns of T, TC2 and TC4. TC*x* represents the TiO_2/$CsPbBr_3$ hybrids, where T and C denote TiO_2 and $CsPbBr_3$ QDs, respectively; *x* represents the weight percentage of $CsPbBr_3$ with respect to TiO_2.

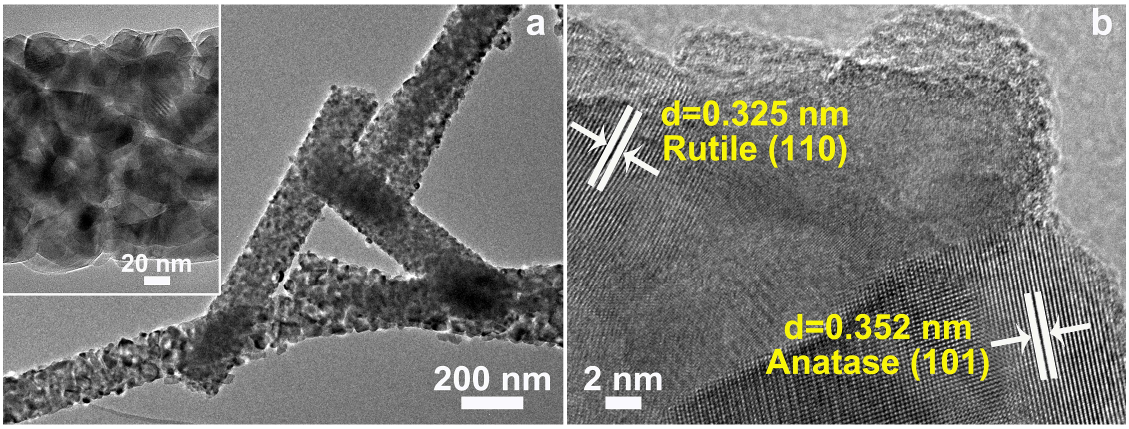

Supplementary Figure 2. Morphology of TiO_2 nanofibers. a TEM image, inset on upper left is the corresponding high-magnification TEM image. **b** HRTEM image.

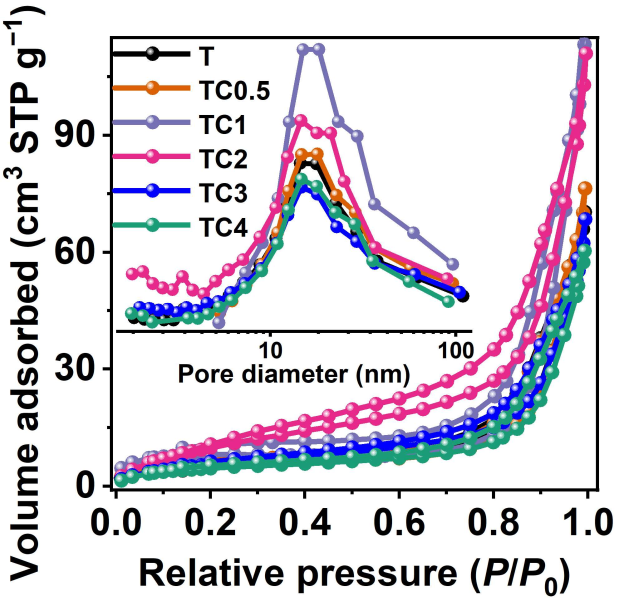

Supplementary Figure 3. Nitrogen adsorption-desorption isotherm and the corresponding pore size distribution (inset) of T, TC0.5, TC1, TC2, TC3 and TC4. TC*x* represents the $TiO_2/CsPbBr_3$ hybrids, where T and C denote TiO_2 and $CsPbBr_3$ QDs, respectively; *x* represents the weight percentage of $CsPbBr_3$ with respect to TiO_2.

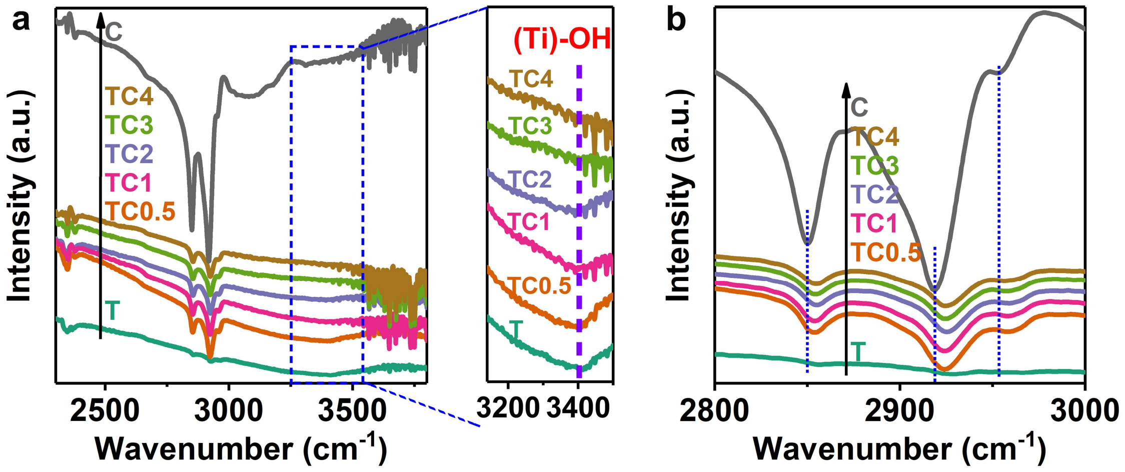

Supplementary Figure 4. Fourier transform infrared (FTIR) spectra of T, TC*x* and C. **a** The wavenumber ranges from 2300 to 3600 cm^{-1}. **b** The wavenumber ranges from 2800 to 3000 cm^{-1}. TC*x* represents the TiO_2/$CsPbBr_3$ hybrids, where T and C denote TiO_2 and $CsPbBr_3$ QDs, respectively; *x* represents the weight percentage of $CsPbBr_3$ with respect to TiO_2.

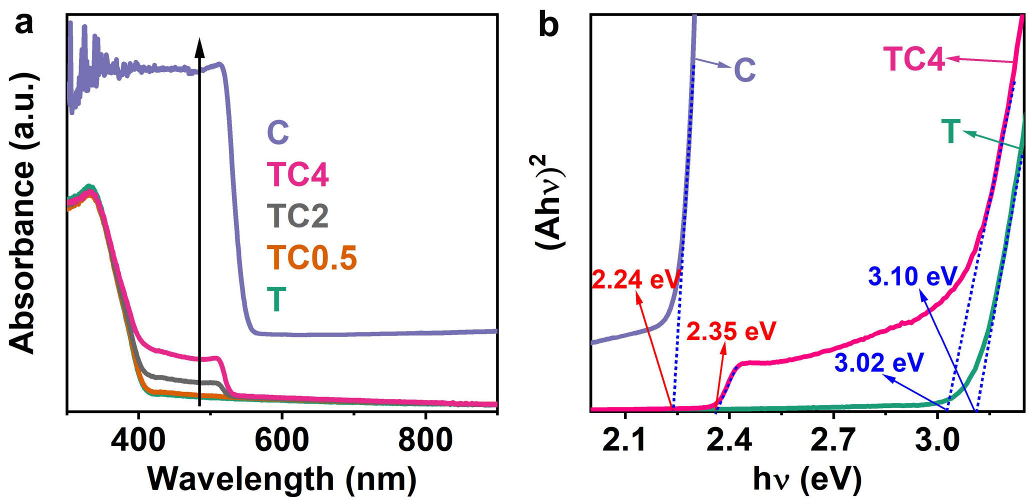

Supplementary Figure 5. Optical properties of resultant samples. a UV-vis diffuse reflectance spectra of T, TC0.5, TC2, TC4 and C. **b** Kubelka–Munk energy curve plots of T, TC4 and C. TC*x* represents the TiO_2/$CsPbBr_3$ hybrids, where T and C denote TiO_2 and $CsPbBr_3$ QDs, respectively; *x* represents the weight percentage of $CsPbBr_3$ with respect to TiO_2.

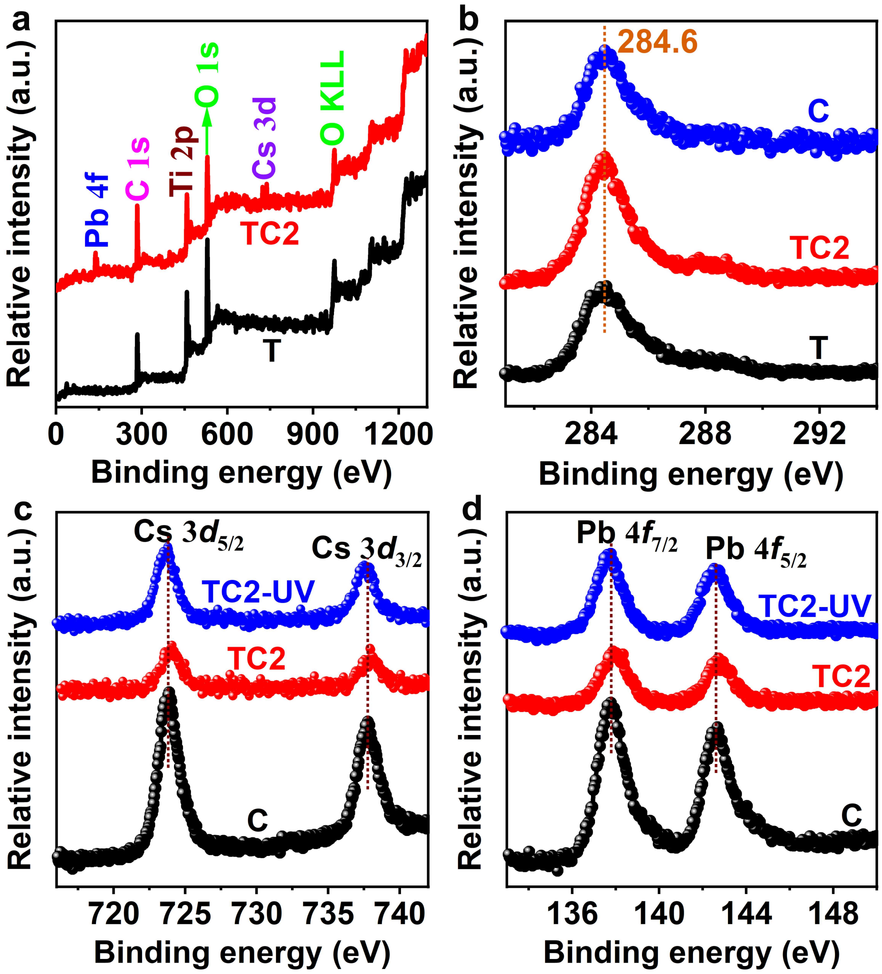

Supplementary Figure 6. X-ray photoelectron spectroscopy (XPS) spectra of resultant samples. **a** The survey XPS spectra of T and TC2. **b** *Ex-situ* XPS spectra of C 1*s* of T, TC2 and C. *In-situ* and *ex-situ* XPS spectra **c** Cs 3*d* and **d** Pb 4*f* of T, TC2 and C. *In-situ* XPS spectra were recorded under UV-vis light irradiation. TC2 represents the $TiO_2/CsPbBr_3$ hybrid, where T and C denote TiO_2 and $CsPbBr_3$ QDs, respectively; 2 represents the weight percentage of $CsPbBr_3$ with respect to TiO_2.

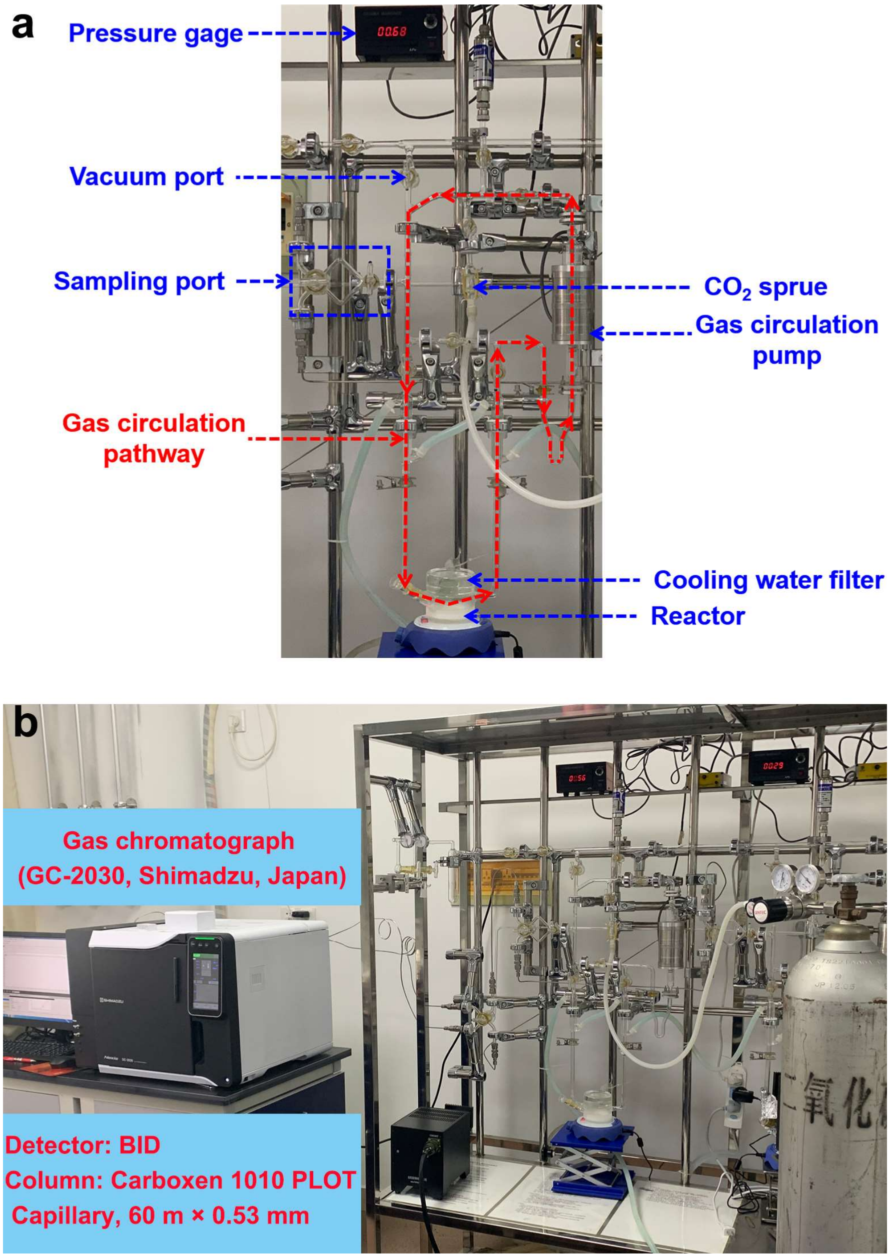

Supplementary Figure 7. The closed gas circulation system for photocatalytic CO_2 reduction reaction. a The gas circulation pathway during the CO_2 photoreduction. **b** The whole online closed gas circulation system.

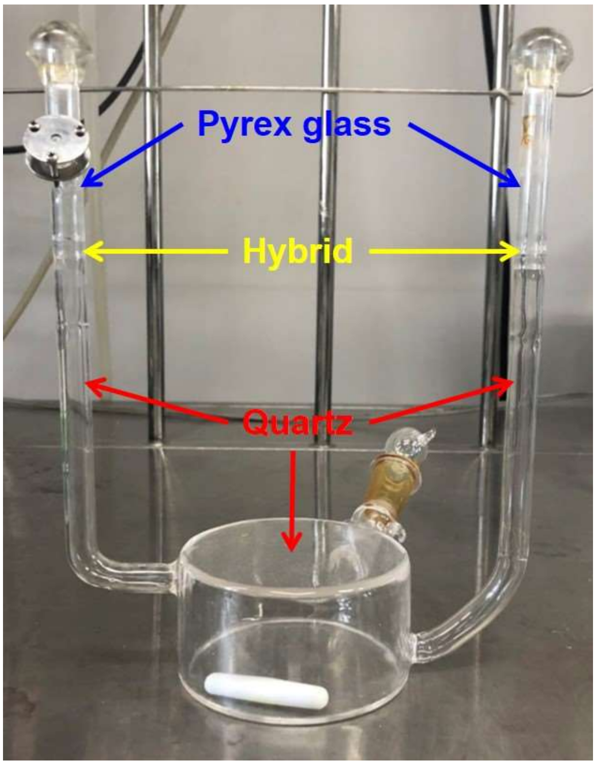

Supplementary Figure 8. The Quartz and Pyrex glass hybrid reaction cell for photocatalytic CO_2 conversion.

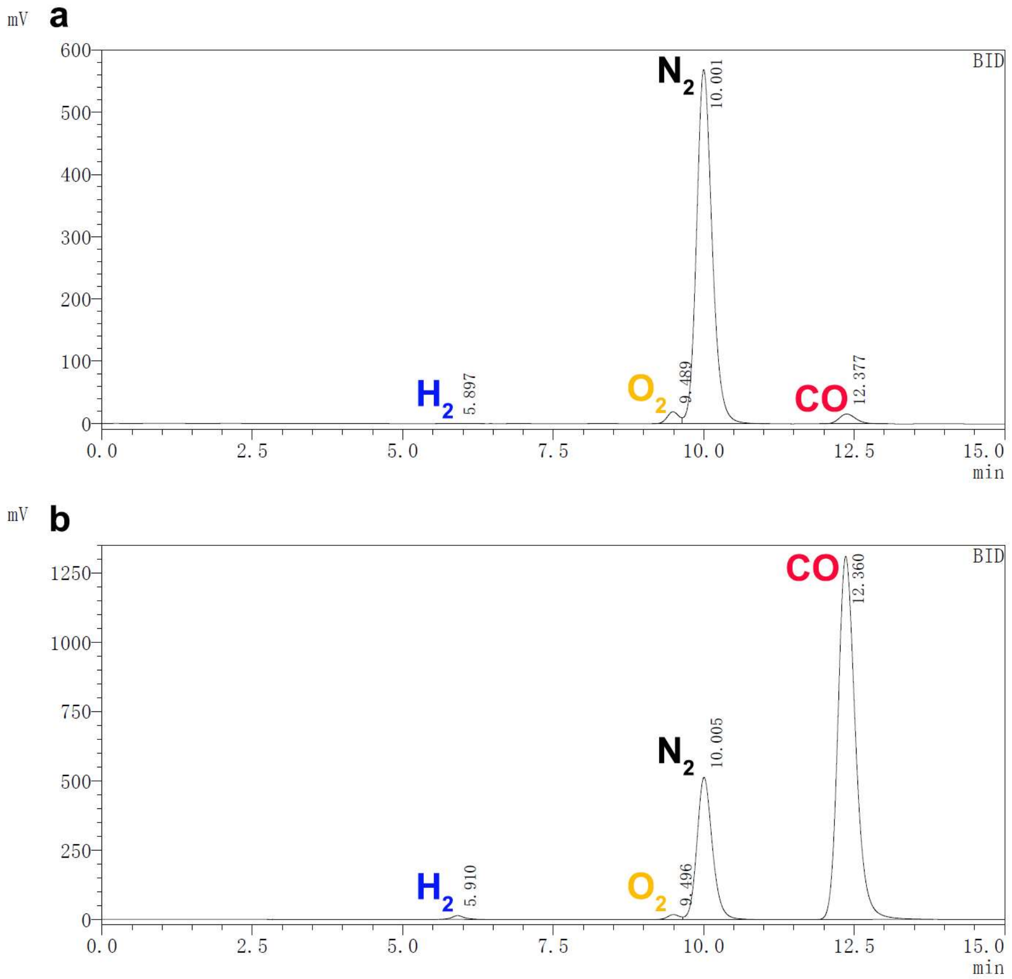

Supplementary Figure 9. The original chromatograms for the reduction of CO_2 on sample TC2. a Without [Ru^{II}(bpy)$_3$]$Cl_2 \cdot 6H_2O$ and BIH. **b** With [Ru^{II}(bpy)$_3$]$Cl_2 \cdot 6H_2O$ and BIH. TC2 represents the $TiO_2/CsPbBr_3$ hybrid, where T and C denote TiO_2 and $CsPbBr_3$ QDs, respectively; 2 represents the weight percentage of $CsPbBr_3$ with respect to TiO_2.

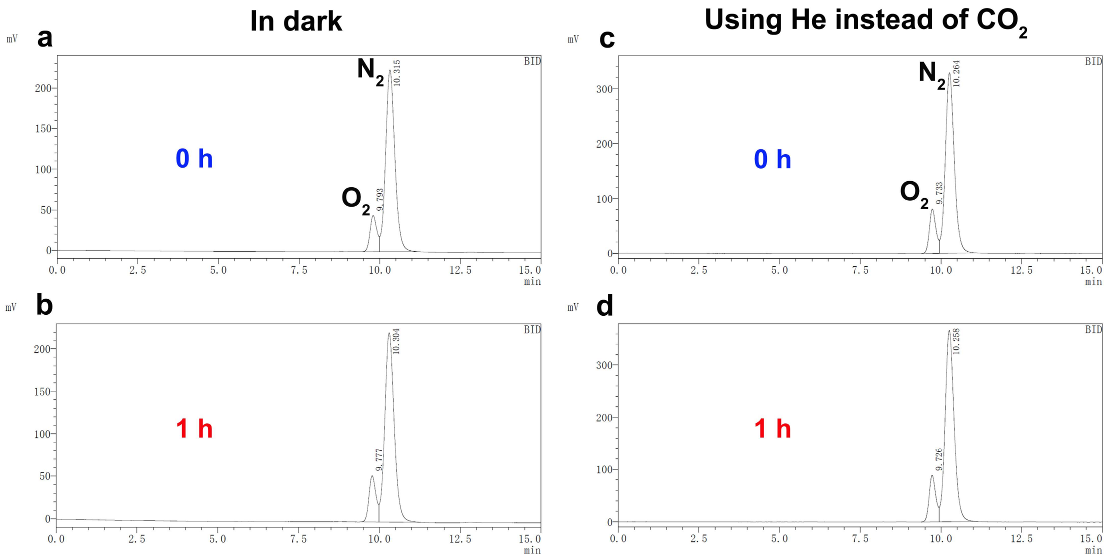

Supplementary Figure 10. The original chromatograms of TC2. **a, b** In dark; **c, d** Using He instead of CO_2 before and after 1 h irradiation. TC2 represents the TiO_2/$CsPbBr_3$ hybrid, where T and C denote TiO_2 and $CsPbBr_3$ QDs, respectively; 2 represents the weight percentage of $CsPbBr_3$ with respect to TiO_2.

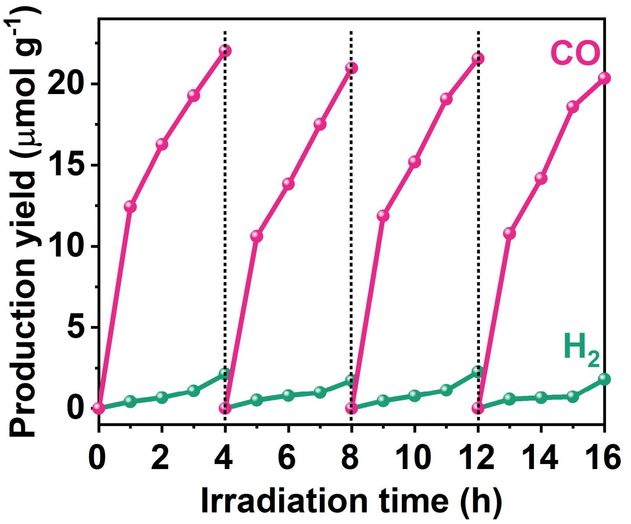

Supplementary Figure 11. The recyclability of TC2 for the photocatalytic CO_2 reduction reaction. TC2 represents the TiO_2/$CsPbBr_3$ hybrid, where T and C denote TiO_2 and $CsPbBr_3$ QDs, respectively; 2 represents the weight percentage of $CsPbBr_3$ with respect to TiO_2.

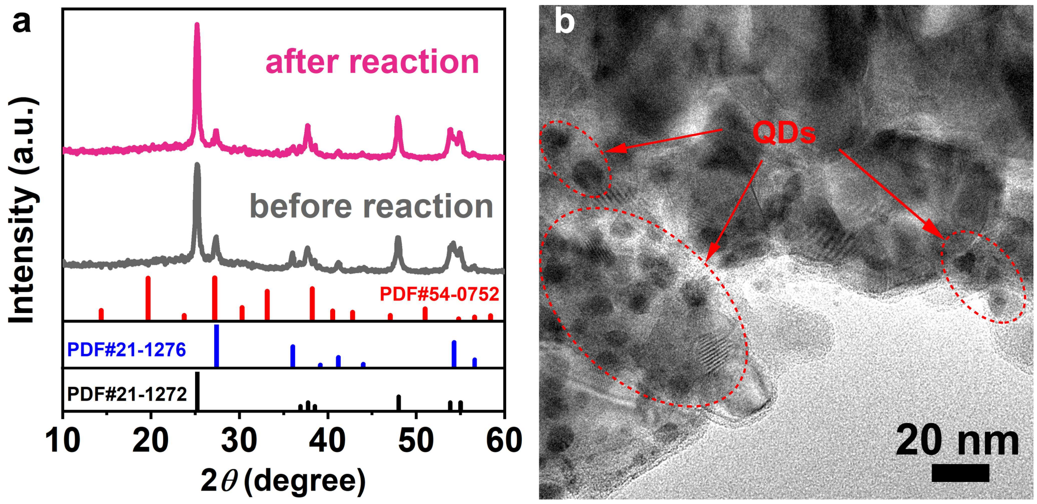

Supplementary Figure 12. Evaluation of the photostability of TC2. a XRD patterns of TC2 before and after reaction. **b** TEM image of TC2 after photocatalytic reaction. TC2 represents the $TiO_2/CsPbBr_3$ hybrid, where T and C denote TiO_2 and $CsPbBr_3$ QDs, respectively; 2 represents the weight percentage of $CsPbBr_3$ with respect to TiO_2.

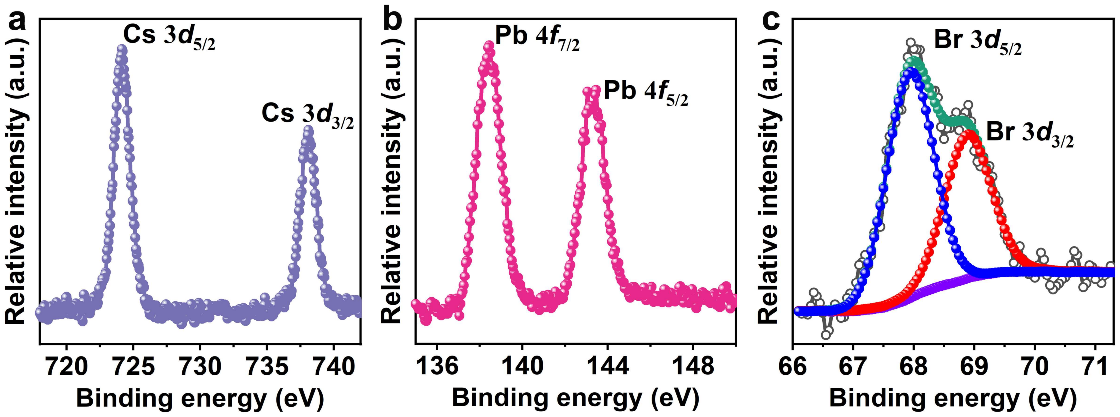

Supplementary Figure 13. *Ex-situ* XPS spectra of **a** Cs 3*d*, **b** Pb 4*f* and **c** Br 3*d* of TC2 after photocatalytic reaction. TC2 represents the $TiO_2/CsPbBr_3$ hybrid, where T and C denote TiO_2 and $CsPbBr_3$ QDs, respectively; 2 represents the weight percentage of $CsPbBr_3$ with respect to TiO_2.

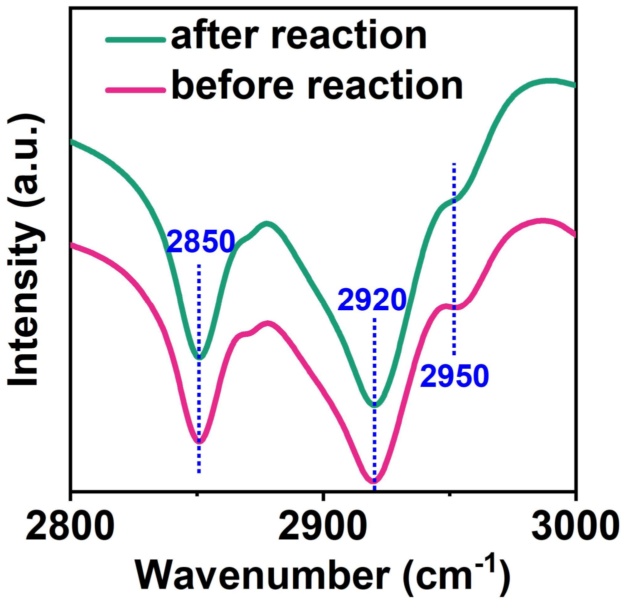

Supplementary Figure 14. The FTIR spectra of TC2 before and after photocatalytic reaction. TC2 represents the $TiO_2/CsPbBr_3$ hybrid, where T and C denote TiO_2 and $CsPbBr_3$ QDs, respectively; 2 represents the weight percentage of $CsPbBr_3$ with respect to TiO_2.

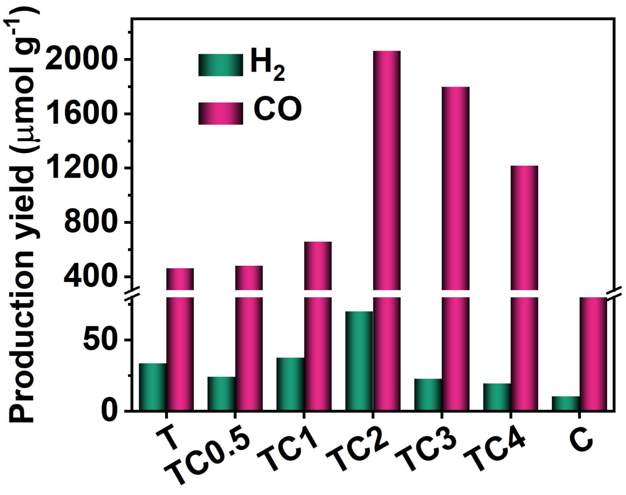

Supplementary Figure 15. Photocatalytic activities of CO_2 reduction over T, TC*x* and C with the help of $[Ru^{II}(bpy)_3]Cl_2 \cdot 6H_2O$ and BIH. TC*x* represents the $TiO_2/CsPbBr_3$ hybrids, where T and C denote TiO_2 and $CsPbBr_3$ QDs, respectively; *x* represents the weight percentage of $CsPbBr_3$ with respect to TiO_2.

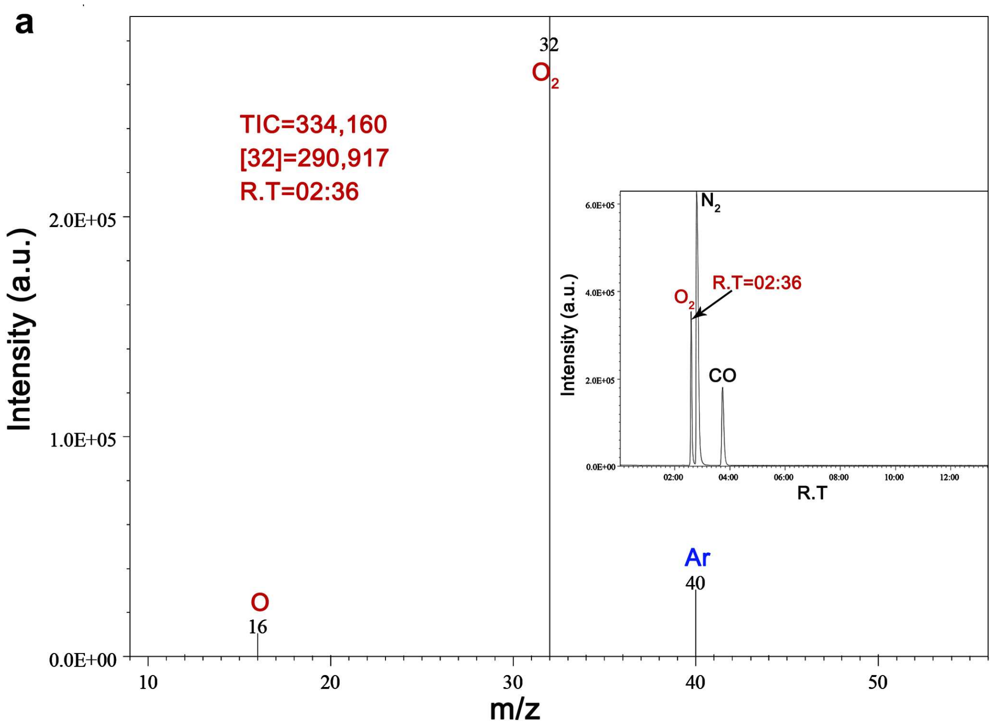

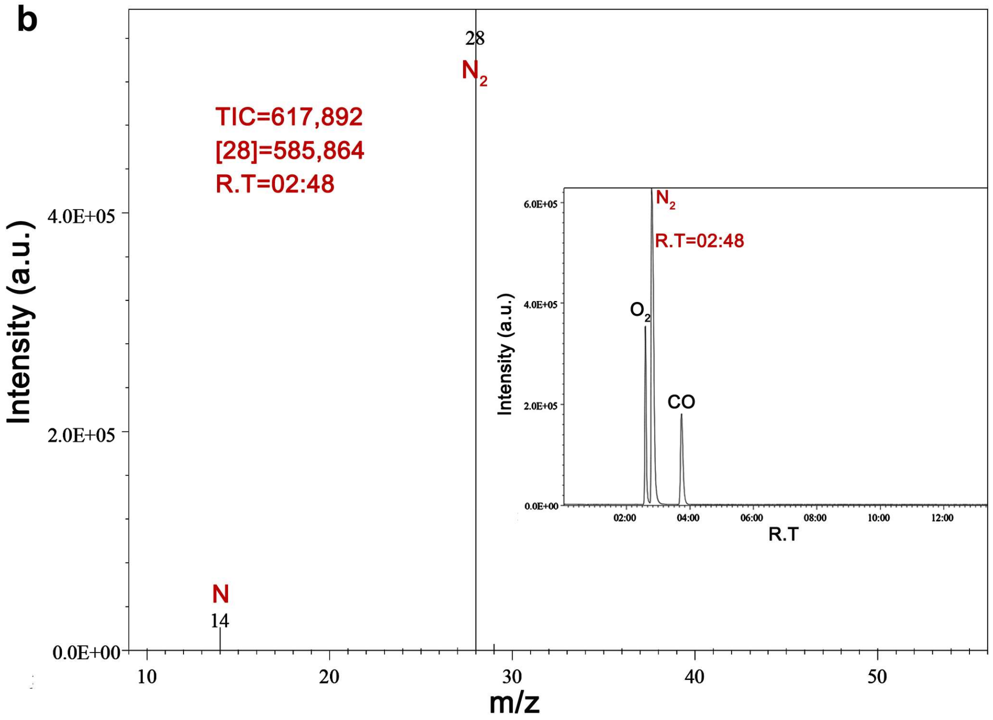

Supplementary Figure 16. Mass spectra and total ion chromatography (inset) of **a** O_2, **b** N_2 over TiO_2/$CsPbBr_3$ heterojunction in the photocatalytic reduction of $^{13}CO_2$.

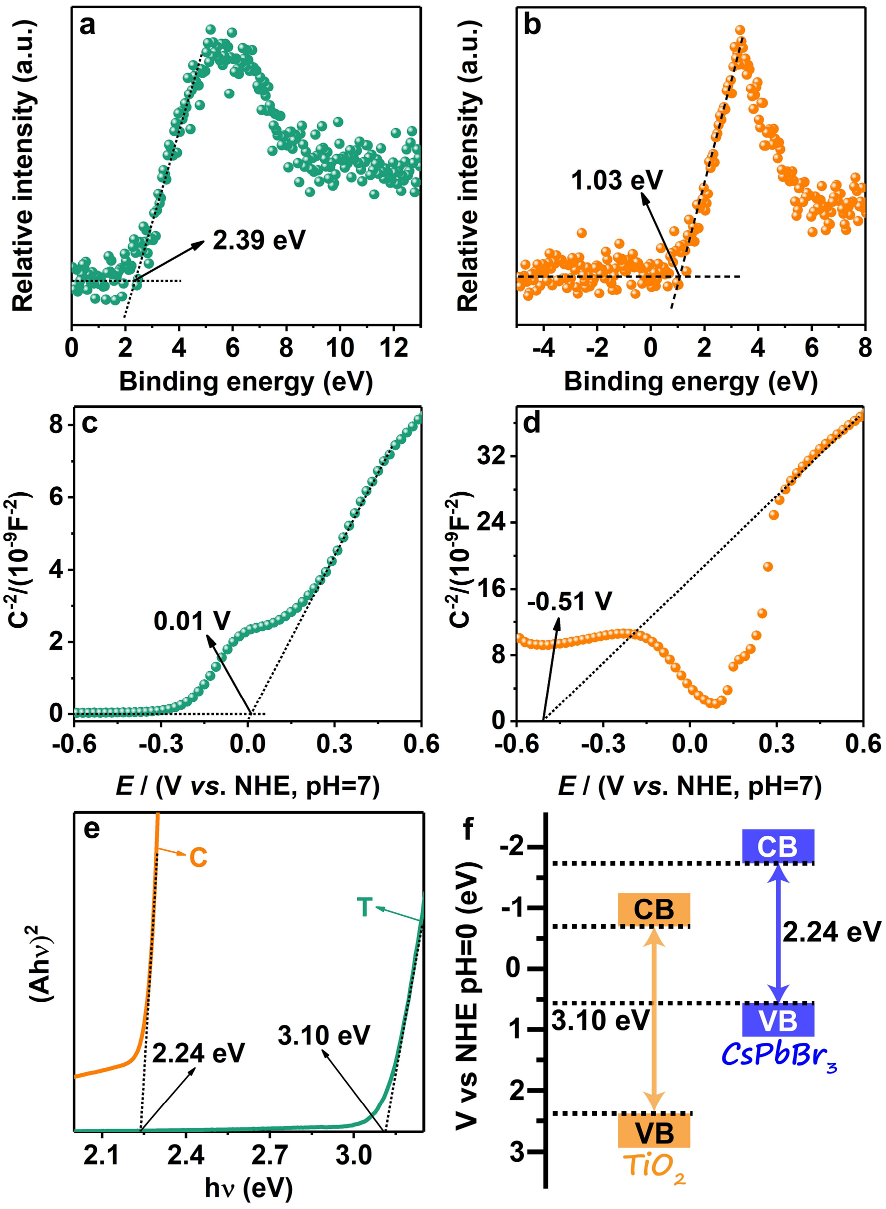

Supplementary Figure 17. Band structures of TiO_2 and $CsPbBr_3$ QDs. Valence band (VB) XPS spectra of **a** TiO_2 and **b** $CsPbBr_3$ QDs. Mott-Schottky plots of **c** TiO_2 and **d** $CsPbBr_3$ QDs. **e** Kubelka–Munk energy curve plots of TiO_2 and $CsPbBr_3$ QDs. **f** Band structures of the composite photocatalyst.

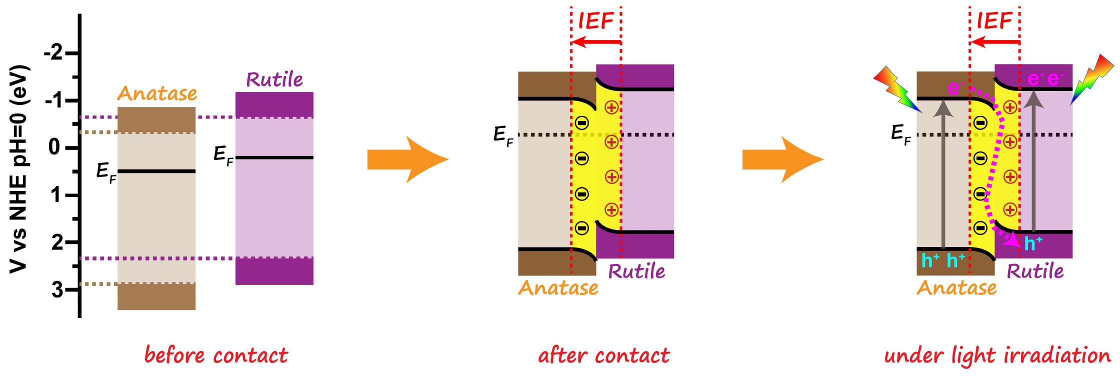

Supplementary Figure 18. Schematic illustration of anatase/rutile homojunction: internal electric field-induced charge transfer, separation and the formation of S-scheme heterojunction under UV-visible light irradiation.

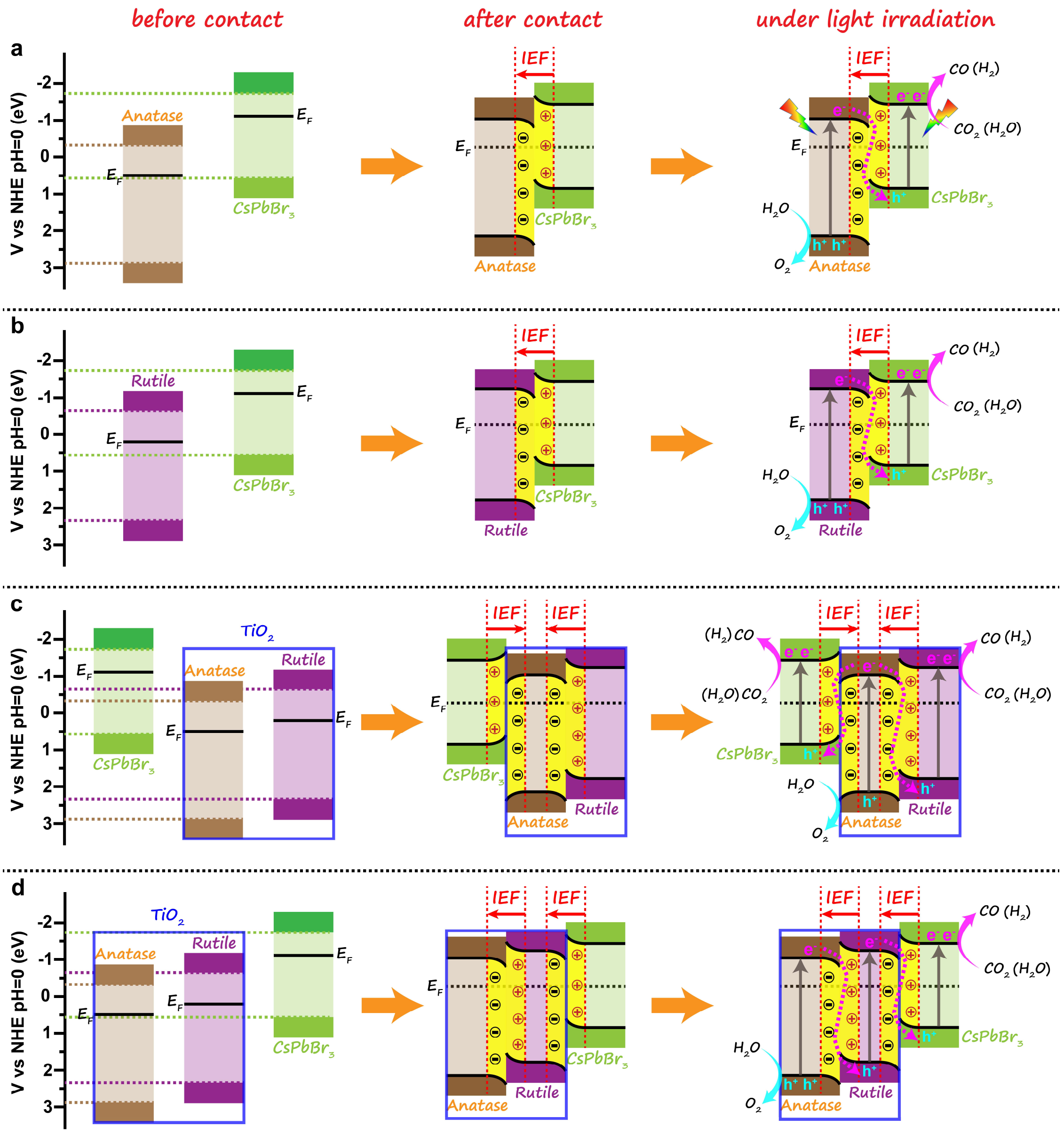

Supplementary Figure 19. Possible schematic illustrations of TiO_2/$CsPbBr_3$ heterojunction. **a** $CsPbBr_3$ QDs contacted with anatase TiO_2, **b** $CsPbBr_3$ QDs contacted with rutile TiO_2, **c** $CsPbBr_3$ QDs contacted with anatase TiO_2 and anatase TiO_2 contacted with rutile TiO_2, **d** $CsPbBr_3$ QDs contacted with rutile TiO_2 and rutile TiO_2 contacted with anatase TiO_2.

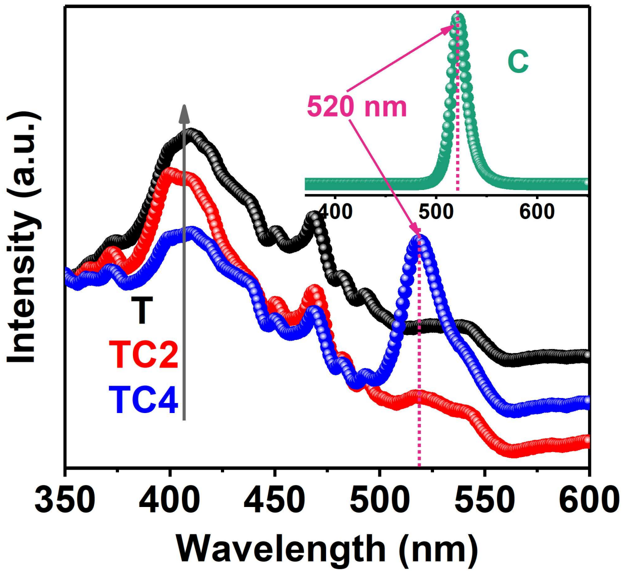

Supplementary Figure 20. Photoluminescence (PL) spectra of T, TC2, TC4 and C. TC*x* represents the $TiO_2/CsPbBr_3$ hybrids, where T and C denote TiO_2 and $CsPbBr_3$ QDs, respectively; *x* represents the weight percentage of $CsPbBr_3$ with respect to TiO_2.

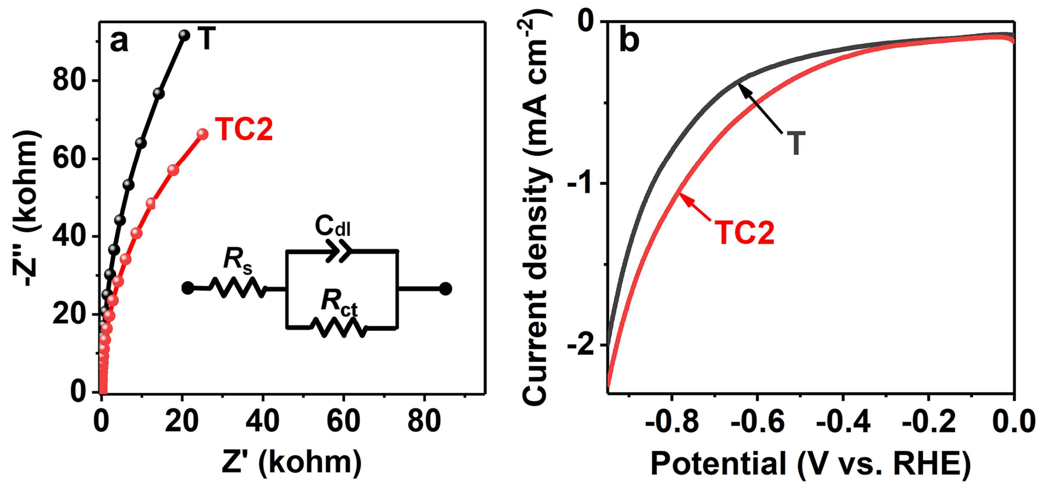

Supplementary Figure 21. Electrochemical Characterization. a Nyquist plots of T and TC2 in 0.5 M Na_2SO_4 aqueous solution. **b** Polarization curves of T and TC2 at a scan rate of 5 mV s^{-1} in 0.5 M Na_2SO_4 under UV-visible light irradiation. TC2 represents the $TiO_2/CsPbBr_3$ hybrid, where T and C denote TiO_2 and $CsPbBr_3$ QDs, respectively; 2 represents the weight percentage of $CsPbBr_3$ with respect to TiO_2.

Supplementary Tables

Supplementary Table 1. Physical properties of the samples with different $CsPbBr_3$ QDs loadings. TC*x* represents the $TiO_2/CsPbBr_3$ hybrids, where T and C denote TiO_2 and $CsPbBr_3$ QDs, respectively; *x* represents the weight percentage of $CsPbBr_3$ with respect to TiO_2.

**Samples**	S_{BET} **(m² g⁻¹)**	V_p **(m³ g⁻¹)**	d_p **(nm)**
T	19	0.09	17.6
TC0.5	21	0.09	18.4
TC1	28	0.14	20.2
TC2	42	0.15	17.1
TC3	22	0.08	14.5
TC4	18	0.07	13.0

S_{BET}: specific surface area, V_p: pore volume, d_p: average pore size

Supplementary Table 2. H_2 and CO production yields over TC2 after UV-vis light irradiation for one hour of various controlled experiments. TC2 represents the TiO_2/$CsPbBr_3$ hybrid, where T and C denote TiO_2 and $CsPbBr_3$ QDs, respectively; 2 represents the weight percentage of $CsPbBr_3$ with respect to TiO_2.

	H_2 (μmol)	CO (μmol)
[a]	0.004	0.13
[b]	0.70	20.65
[c]	0.04	4.81
[d]	Not determined	Not determined
[e]	Not determined	Not determined
[f]	0.013	0.045
[g]	0.003	0.026

[a]: 10 mg catalyst+30 mL acetonitrile+100 μL H_2O;

[b]: 10 mg catalyst+2 mM $[Ru^{II}(bpy)_3]Cl_2\cdot6H_2O$+10 mM BIH+30 mL acetonitrile+100 μL H_2O;

[c]: 2 mM $[Ru^{II}(bpy)_3]Cl_2\cdot6H_2O$+10 mM BIH+30 mL acetonitrile+100 μL H_2O;

[d]: in dark;

[e]: using He instead of CO_2;

[f]: 10 mg catalyst+10 mM BIH+30 mL acetonitrile+100 μL H_2O;

[g]: 10 mg catalyst+2 mM $[Ru^{II}(bpy)_3]Cl_2\cdot6H_2O$+30 mL acetonitrile+100 μL H_2O.

Supplementary Table 3. The fitted parameters obtained from decay curves of the samples. TC2 represents the $TiO_2/CsPbBr_3$ hybrid, where T and C denote TiO_2 and $CsPbBr_3$ QDs, respectively; 2 represents the weight percentage of $CsPbBr_3$ with respect to TiO_2.

**Sample**	E_W	τ_1 **(ns) (Rel. %)**	τ_2 **(ns) (Rel. %)**	τ_3 **(ns) (Rel. %)**	τ_a **(ns)**	χ^2
**T**	450 nm	0.56 (37.98)	2.99 (46.82)	13.80 (15.20)	1.3	1.01
**TC2**	450 nm	0.82 (36.27)	3.56 (51.32)	19.36 (12.41)	2.1	1.13
**TC2**	520 nm	1.76 (30.12)	6.40 (39.95)	40.38 (29.94)	10.4	1.07

τ_a represents the average lifetime (ns) of photogenerated carriers; τ_1, τ_2 and τ_3 represent the lifetime (ns) in the radiative, non-radiative and energy transfer process, respectively; χ^2 represents the goodness of fit parameter.

Supplementary Methods

Computational details. The density functional theory (DFT) calculations were carried out by using the CASTEP module. The exchange–correlation interaction was described by generalized gradient approximation (GGA) with the Perdew–Burke–Ernzerhof (PBE) functional. The energy cutoff and Monkhorst–Pack **k**-point mesh were set as 450 eV and 2 × 2 × 1, respectively. During the geometry optimization, the convergence tolerance was set as 1.0×10^{-5} eV atom^{-1} for energy and 0.03 eV Å^{-1} for force. For the construction of surface models, a vacuum of 20 Å was used to eliminate interactions between periodic structures. The work function is defined as $\Phi = E_V - E_F$, where E_V and E_F are the electrostatic potentials of the vacuum and Fermi levels, respectively.
